# An Uncommon Case of Chronic Tubercular Appendicitis

**DOI:** 10.1155/2015/534838

**Published:** 2015-11-16

**Authors:** Sushna Maharjan

**Affiliations:** Department of Pathology, Chitwan Medical College Teaching Hospital, P.O. Box 42, Bharatpur-10, Chitwan, Nepal

## Abstract

Tuberculosis (TB) is a common disease that ranks as the second leading cause of death from an infectious disease worldwide, after the human immunodeficiency virus (HIV). However, primary TB of the appendix is rare and may or may not be associated with specific clinical features. Thus, diagnosis is made only after histopathological examination. It suggests that all surgically removed appendices should be subjected to histopathological examination. This reported case is an uncommon case of chronic tubercular appendicitis.

## 1. Introduction

Tuberculosis (TB) is a major global health problem. The current global scenario of TB shows an estimated 9.0 million incident cases of TB and 1.5 million people who died of the disease in 2013. Among 9.0 million of cases, more than half (56%) were in the Southeast Asia and Western Pacific Regions. Another one-quarter was in the African Region. It accounted for 24% and 11% of total cases, respectively, in India and China alone. In 2013, an estimated 13% of new TB cases were HIV-positive [1]. The incidence of TB is rising in developed countries because of acquired immune deficiency syndrome (AIDS) and it indicates a global comeback of TB in combination with AIDS [2].

Terminal ileum [3, 4], ileocaecal junction [5], and peritoneum [2] are the most common sites of TB in abdomen. It is quite interesting that involvement of the appendix is very rare although it lies so close to ileocecal region. Primary appendicular TB as isolated form is even rarer [6].

Most of the patients with gastrointestinal (GI) TB commonly present with the clinical features of abdominal pain, fever, and weight loss [2]. However, the diagnosis may be difficult due to nonspecific clinical presentation. Patients who have appendicular TB may present with signs and symptoms similar to acute appendicitis and make it difficult for a correct diagnosis [6, 7]. Thus, the diagnosis of appendicular TB is usually made on histopathological examination which delays treatment of the patient.

## 2. Case Report

A 40-year-old male presented with chronic abdominal pain for 2 weeks with history of diarrhea and weakness. Ultrasonogram of abdomen diagnosed the case as inflamed appendix. On examination of abdomen, there was tenderness in right iliac fossa with rebound localized to McBurney's point. Total leucocyte count was raised to 16,000/cu mm. Other routine biochemical and hematological investigations were normal. Clinical diagnosis was made as chronic appendicitis and appendectomy was performed. Peroperative findings were inflamed appendix studded with few tubercles. Thus, appendix and mesenteric lymph node were sent for histopathological examination for definite diagnosis.

**Figure 1:**
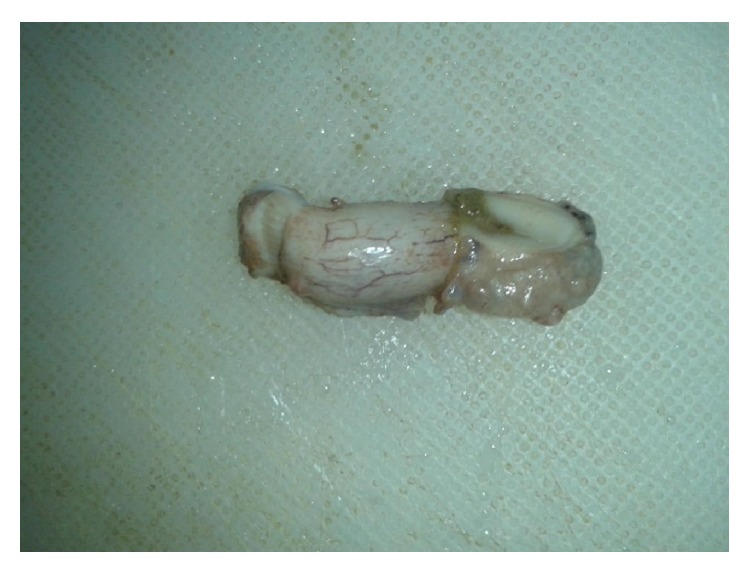
Gross specimen of appendix showing multiple tubercles in periappendiceal fat and surface.

**Figure 2:**
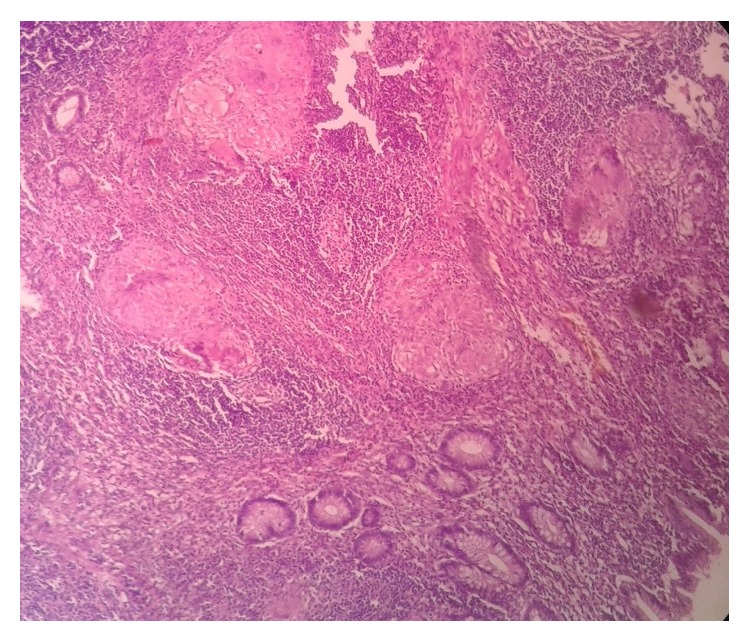
Histopathological examination of appendix showing numerous granulomas in the wall at low power.

**Figure 3:**
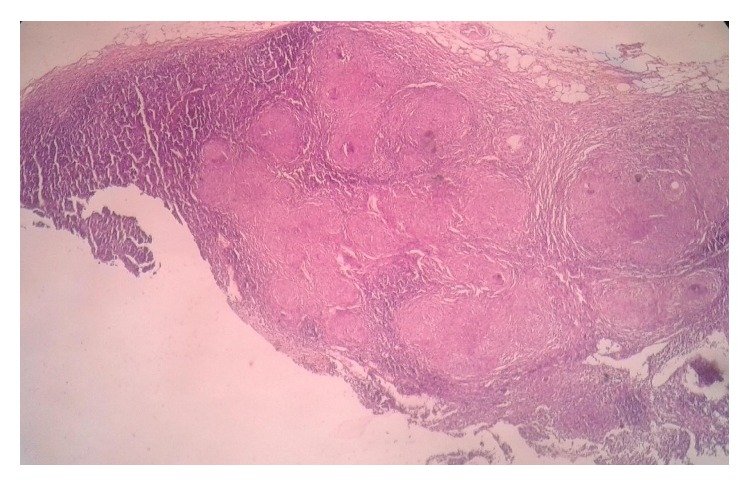
Histopathological examination of mesenteric lymph node with multiple granulomas at low power.

**Figure 4:**
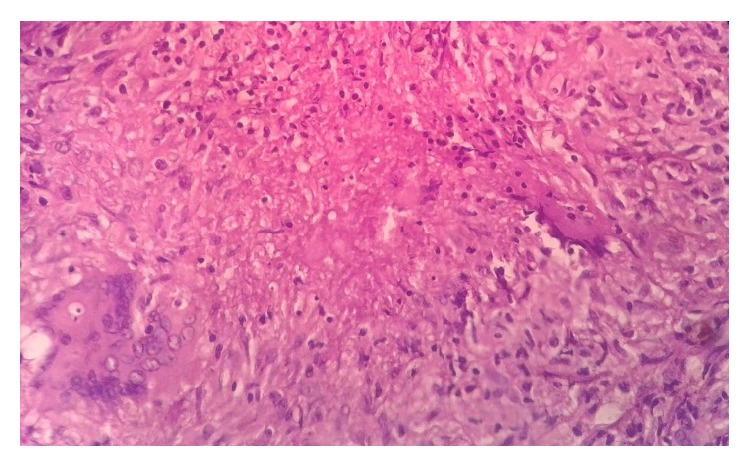
Histopathological examination of appendix showing caseation in the centre with surrounding multinucleated giant cells and epithelioid histiocytes at high power.

Grossly, appendix measured 4.5 × 1 cm. External surface was congested and tubercles were present on the serosal surface and periappendiceal fat (Figure 1). Cut section of appendix showed yellowish foci of caseous necrosis in the wall. Lymph node measured 0.8 × 0.5 cm with focal yellowish area of caseous necrosis likewise in appendix. Histopathological examination revealed transmural inflammation of appendicular wall and also lymph node composed of caseating granulomas with epithelioid histiocytes, Langhans' giant cells, and lymphocytes (Figures 2, 3, and 4). However, tubercle bacilli were not detected by Ziehl-Neelsen (ZN) stain. Diagnosis of tuberculous appendicitis with mesenteric lymph node involvement was given. He was treated with antitubercular therapy in directly observed treatment short course centre (DOTs). The patient was doing well and showed clinical recovery on follow-up in regular intervals. Primary focus was not detected on further investigations of TB involving other organs. HIV was also nonreactive.

## 3. Discussion

Infection of hollow or solid abdominal organs, peritoneum, and abdominal lymphatics with* Mycobacterium tuberculosis* bacilli is known as abdominal TB. The isolated form is less observed than combination of two or more sites [2]. The present case was a combination of GI TB involving appendix and mesenteric lymph node.

Among the abovementioned sites of abdomen (hollow organs), GI TB in patients with active pulmonary TB before the advent of specific antitubercular treatment had been reported as high as 55–90%, but the cases decreased to 25% after specific drugs developed [8]. The success rate of treatment was 86% among all new TB cases globally in 2012 [1].

The estimated incidence of abdominal TB among all cases of TB is 1–3% worldwide [9]. GI TB is more common in developing countries like Nepal [2]. Demographically the incidence of TB appendicitis has also shown variation in different countries. In Brunei Darussalam, it comprised 0.2% of all TB cases and 8.6% of abdominal TB cases among 6,593 appendectomies performed [7]. A study conducted in Nepal reported that cases of granuloma consistent with TB constituted 0.58% of total 518 appendectomy specimens [10]. In a tertiary centre in India, 2.3% of cases of TB appendicitis among 2921 appendectomies were diagnosed, being much higher than the reported cases in Nepal [11]. Tuberculous peritonitis was diagnosed in 2 cases among total cases of 177 abdominal surgeries in the other study of Nepal. It was not explained how tuberculous peritonitis occurred, whether it was manifestations of disseminated TB or if there was perorated GI TB [12].

It was thought that ingestion of milk contaminated with TB bacilli,* Mycobacterium bovis*, was the reason behind primary GI TB but boiled milk is usually consumed in developing countries and pasteurized milk in the west which makes it unlikely for ingestion of bacilli [2]. The reason behind rare involvement of the isolated appendix in spite of the ileocaecal junction being the common site of involvement in intestinal TB is that luminal mucosa of the appendix has minimal exposure with the intestinal contents [13, 14].

Secondary appendicular TB is spread through hematogenous route or swallowing of infected sputum from primary pulmonary TB or direct contact through the infected lymph nodes and retrograde spread via fallopian tube infection [2].

Abdominal TB is common between 25 and 45 years of age [15]. Similarly other studies also found that young adults of 21–40 years are most commonly affected by abdominal TB [3–5]. TB appendix was found in age group of 25–48 years in a study conducted by Chong et al. [7].

Patients present with a vague clinical presentation in GI TB. Abdominal pain, weight loss, diarrhea, and intermittent constipation associated with worsening of the pain were common in a study of Nepal by Gautam et al. [2]. Abdominal pain was the most common presenting symptom followed by fever and weight loss [16] similar to the triad of symptoms in a study of Nepal. Patients also may present with constitutional symptoms. Symptoms of intestinal obstruction such as abdominal pain, constipation, and vomiting are usually seen in intestinal TB [15].

Chong et al. found that majority of patients with TB appendix had symptoms consistent with acute appendicitis [7]. Other symptoms such as localized or generalized ascites, abdominal distention, diarrhea, melaena, and anemia may be seen [16]. Hence, we cannot point out specific clinical features that are pathognomonic of appendicular TB.

Granulomatous appendicitis has other differential diagnoses such as parasite-related appendicitis, Crohn's disease, sarcoidosis, and foreign body-induced inflammation [11]. Differentiation between Crohn's disease and TB is very important among these conditions because of different approach to the management of these two diseases. Glucocorticosteroid is the main choice of drug for the treatment in Crohn's disease [2]. However, use of steroid therapy for patients with TB was hazardous without antituberculous therapy [17], but utility of adjunctive corticosteroid therapy in the management of TB with effective antituberculous drugs could improve outcomes [18–20]. It has proved to be beneficial in miliary TB, tuberculous meningitis, tuberculous pericarditis, tuberculous pleurisy, and endobronchial TB [21, 22]. The improvement of the outcome in TB patients with corticosteroids therapy is by suppressing the host mediated inflammation. When steroid therapy is given to patients with untreated or unrecognized TB, it results in overwhelming disease and death. Hence, clinicians must closely observe such patients and one should always remember drug interactions that may occur during treatment [22].

## 4. Conclusion

The present case was not suspected as TB of appendix unless laparotomy was performed and suspected tubercles were noticed. Diagnosis was made only after histopathological examination. Hence, it suggests that all surgically removed appendices should be subjected to histopathological examination.
